# Clinicopathological and Prognostic Significance of CD47 Expression in Lung Neuroendocrine Tumors

**DOI:** 10.1155/2021/6632249

**Published:** 2021-06-11

**Authors:** Mario Orozco-Morales, Alejandro Avilés-Salas, Norma Hernández-Pedro, Rodrigo Catalán, Graciela Cruz-Rico, Ana Laura Colín-González, Elsa Dosal-Mancilla, Pedro Barrios-Bernal, Oscar Arrieta

**Affiliations:** ^1^Thoracic Oncology Unit and Personalized Medicine Laboratoy, Instituto Nacional de Cancereología, Mexico City, Mexico; ^2^Pathology Department, Instituto Nacional de Cancerología (INCan), Mexico City, Mexico

## Abstract

**Background:**

Lung neuroendocrine tumors account for approximately 15% of all lung cancer cases. LNET are subdivided into typical carcinoid (TC), atypical carcinoid (AC), large cell neuroendocrine carcinoma (LCNEC), and small-cell lung cancer (SCLC). The Ki-67 index has been used for decades to evaluate mitotic counts however, the role of Ki-67 as a biomarker for assessing prognosis and guiding therapy in metastatic LNET still lacks feasible clinical validation. Recent clinical trials have indicated that inhibition of CD47 with anti-CD47 antibodies exerts a promising antitumor effect against several human malignancies, including NSCLC, melanoma, and hematologic malignancies. However, the clinical relevance of CD47 expression in LNET has remained unclear.

**Methods:**

We performed a retrospective study in which we analyzed tumor biopsies from 51 patients with a confirmed diagnosis of LNET that received treatment at our hospital. Then, we analyzed if there was any correlation between CD47 expression with any clinical or pathological characteristic. We also analyzed the prognostic significance of CD47, assessed as progression-free survival and overall survival.

**Results:**

A total of 51 patients with LNET were enrolled in our study. The mean age at diagnosis was 57.6 (±11.6) years; 30 patients were women (59%). 27.5% of patients were positive for CD47 expression, and 72.5% of patients showed a CD47 expression of less than 1% and were considered as negatives. In patients with high-grade tumors (this time defined as Ki‐67 > 40%), the positive expression of CD47 was strongly associated with an increased PFS. Albeit, these differences did not reach statistical significance when analyzing OS.

**Conclusion:**

Contrary to what happens in a wide range of hematologic and solid tumors, a higher expression of CD47 in patients with LNET is associated with a better progression-free survival, especially in patients with a Ki‐67 ≥ 40%. This “paradox” remains to be confirmed and explained by larger studies.

## 1. Introduction

Lung neuroendocrine tumors (LNET) are account for approximately 15% of all lung cancer cases [1, 2]. LNET are subdivided into typical carcinoid (TC), atypical carcinoid (AC), large cell neuroendocrine carcinoma (LCNEC), and small-cell lung cancer (SCLC) [3]. Classification criteria include mitotic count, the presence or absence of necrosis, cytological and histological characteristics including cell size and shape, nuclear features, and architecture. Clinically, TC are low-grade malignant tumors, AC intermediate-grade malignant tumors, and SCLC/LCNEC high-grade malignant carcinomas that grow rapidly and metastasize early [4, 5]. The Ki-67 index has been used for decades for evaluating mitotic counts; however, the role of Ki-67 as a biomarker for assessing prognosis and guiding therapy in metastatic LNET still lacks feasible clinical validation [6].

Systemic chemotherapy is the current standard of care for patients with LNET who have advanced disease. Although, initially, some LNET show good response to treatment, most patients suffer from disease recurrence and become refractory to chemotherapy; accordingly, the prognosis f patients with LNET is poor, with a 5-year survival rate of less than 5% [7]. In recent years, immunotherapy changed the scenario for LNET; owing to a high immunogenicity, a high tumor mutation burden, and other favorable immune factors, immune checkpoint inhibitors (ICIs) could become a breakthrough in the treatment of LNET [8–10].

CD47 is a cell surface protein which is normally expressed at low levels in every healthy cell. One of the main physiologic function is to act as an inhibitor of phagocytosis; this occurs throughout interaction with signal regulatory protein *α* (SIRP*α*) expressed on macrophages; accordingly, loss of CD47 leads to phagocytosis of aged or damaged cells. However, CD47 is also implicated in other functions including proliferation, apoptosis, and cell-extracellular matrix via ligation with thrombospondins [11, 12].

CD47 was first identified as a tumor antigen on human ovarian cancer, since then CD47 has been found to be overexpressed on multiple hematologic and nonhematologic malignancies, including chronic myeloid leukemia, non-Hodgkin's lymphoma [13], multiple myeloma [14], breast cancer [15], non-small-cell lung cancer (NSCLC) [16, 17], and pancreatic cancer [18]. Increased expression of CD47 on tumors allows malignant cells to escape innate immune surveillance through evasion of phagocytosis by interacting with SIRP*α* on myeloid cells. In this way, cancer cells exploit the “don't eat me signal” provided by CD47 and avoid a cornerstone component of antitumor immune response [19].

Recent clinical trials have indicated that inhibition of this pathway with anti-CD47 antibodies exerts a promising antitumor effect against several human malignancies, including NSCLC, melanoma, and hematologic malignancies [20, 21]. However, the clinical relevance of CD47 expression in LNET has remained unclear.

The aims of the present study were to determine if the expression of CD47 in LNET has prognostic relevance and to determine if the expression of CD47 is associated with other clinical and/or histological characteristics.

## 2. Materials and Methods

### 2.1. Patients and Study Design

We performed a retrospective study in which we analyzed tumor biopsies from 51 patients with a confirmed diagnosis of LNET that received treatment at our hospital (*Instituto Nacional de Cancerolog*ía, Mexico City Mexico) between March 2012 and September 2020. Patients were included if they fulfilled the following criteria: >18 years old, histology confirmation of LNET, Eastern Cooperative Oncology Group Performance Status (ECOG PS) ≤ 2, and disease stage IIIB-IV at the time of tumor biopsy. Patients were excluded if available tissue was not enough to perform IHC assays as planned. The clinical and pathological characteristics that were collected from medical records were: age, gender, smoking history, stage of disease, histological subtype, and site of metastases (if any); all relevant data were collected from electronic medical charts. The entire protocol was performed in accordance with the ethical standards of the Institutional Review Board as well as the Ethical Committee of INCan (011/018/ICI-CV/683) and in accordance with the Declaration Helsinki of 1975.

### 2.2. Immunohistochemistry for CD47 and Ki-67

For IHC analyses, tissue sections of 5 *μ*m formalin-fixed paraffin-embedded samples were deparaffinized and blocked for endogenous peroxidase activity with hydrogen peroxide. Then, antigen retrieval was performed with immune heat-DNA retriever citrate (BSB 0023, Bio SB, Inc.). Samples were washed with 1X Tris-buffered saline (TBS Automation Wash Buffer, 40x) and incubated with an anti-CD47 antibody (clone: B6H12, 1 : 50, 12730; Santa Cruz Biotechnology) and Ki-67 (Ki-67 antibody; 15580; Abcam) at room temperature during 45 minutes. The reaction was visualized using MACH 4 universal HRP-polymer kit (M1U539, Biocare) following incubation with diaminobenzidine for 3 minutes. Afterwards, sections were counterstained with hematoxylin and ammonium hydroxide. Isotype-matched IgG was used as a control for staining, and prostate tissue was used as a positive control. A blinded examination process was performed by an independent pathologist. CD47 staining intensity and the percentage of stained cells were reported by a senior pathologist.

Staining intensity of the cell membrane was divided into 4 categories as follows: no staining, 0; weak staining, 1+ (light brown membrane staining); intermediate staining, 2+; and strong staining, 3+ (dark brown linear membrane staining). For more reliable scoring definitions, strong staining (3+) was clearly visible using a 4x objective lens, moderate staining (2+) required a 10x objective lens for clear observation, and weak staining (1+) required a 40x objective lens. Multiplication of intensity of staining and percentage of immunoreactive cells resulted in an immunoreactivity scoring system, ranging from 0 to 300 for each individual case.

### 2.3. Statistical Analysis

For descriptive purposes, continuous data were summarized as arithmetic means with standard deviation (SD). Data distribution was assessed by the Kolmogorov-Smirnov test. Comparison between groups was performed by Student's *T*-test or Mann-Whitney *U*-test, depending on data distribution. Data from contingency tables was analyzed using a Chi-squared test or Fisher's exact test. Overall survival (OS) and progression-free survival (PFS) were estimated by the Kaplan-Meier method; comparisons among groups were analyzed with the Log-rank test. For survival-curve analysis, all variables were dichotomized (for age, median was used). Adjustment for potential confounders was addressed with a multivariate Cox regression analysis, and hazard ratios were estimated along with their corresponding 95% confidence intervals (CI). A two-sided *p* value of ≤0.05 was considered statistically significant. Data were analyzed with the SPSS software package version 26 (SPSS, IBM, Inc., Chicago, IL, USA).

## 3. Results

### 3.1. Baseline Clinical Characteristics

A total of 51 patients with LNET were enrolled in our study. The mean age at diagnosis was 57.6 (±11.6) years. 30 patients were women (59%). Approximately 60% of patients were smokers, with a mean tobacco index of 48.2 (±39.2). Most patients presented ECOG PS < 2 at the time of diagnosis (78.4%). The most frequent histological subtype was SCLC which was identified in 29 patients (56.9%). According to the Ki-67 index, 74.5% of the cases were classified as high grade and 25.5% were classified as low grade. 72.5% of patients presented stage IV disease, while the other 27.5 (14%) presented stage IIIB. Other baseline characteristics of our population are presented in Table 1.

**Figure 1:**
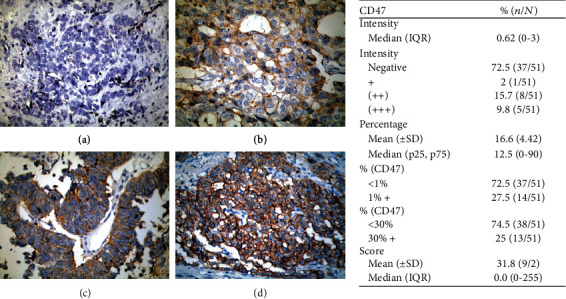
Expression of CD47 in the entire population (51 patients). Images exemplifying negative CD47 expression (a), + CD47 expression (b), ++ CD47 expression (c), and +++ CD47 expression (d).

Immunohistochemical analysis was performed to evaluate the protein expression level of CD47 in the tissues of LNET and adjacent normal mucosa. IHC staining showed that CD47 was primarily located in cytoplasmic membrane of tumor cells and to a lesser extent diffusely in cytoplasm (Figure 1). However, there was a significant variability in staining intensity and percentage of positive cells among patients. 27.5% of patients were positive for CD47 expression, and 72.5% of patients showed a CD47 expression of less than 1% and were considered as negatives.

### 3.2. Association of CD47 Scores with Clinical Characteristics

Based on staining index scores, 51 patients were stratified into two groups: those with CD47 negative (-) (HScore < 30) (*n* = 37) and those with CD47 positive (+) (*n* = 14). Table 1 shows the baseline characteristics of LNET patients according to CD47 status. No differences in baseline characteristics were found among the group with high expression of CD47 and the group with lower expression (Table 1).

### 3.3. Progression-Free Survival and Overall Survival

**Figure 2:**
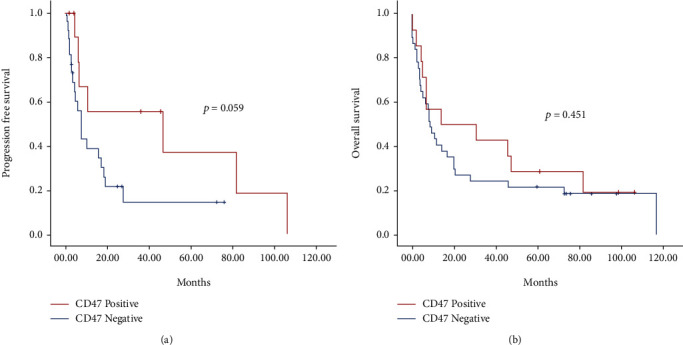
PFS (a) and OS (b) according to CD47 expression of the entire population.

Median progression-free survival (PFS) for first-line therapy was 10.3 months (95% CI 6.3-14.3 months). When analyzing between positive and negative CD47 expression, at the univariate analysis, PFS almost reached statistical significance for a better PFS in the positive group (Table 2; Figure 2(a)).

While analyzing for OS, we found that male gender, smoking history, disease stage IV, pleural effusion, CNS metastases, and liver metastases were all associated with a decreased OS. On the other hand, atypical histology subtype was associated with a better OS (Table 3). When dividing the cohort according to positive or negative expression of CD47, we did not find significant differences among groups (*p* = 0.451) (Figure 2(b)).

### 3.4. Prognosis in Patients with Positive CD47 and Ki‐67 ≥ 40%

In a subanalysis, we found that in patients with high-grade tumors (defined as Ki‐67 > 40%), positive expression of CD47 was strongly associated with a decreased PFS, being 4.83 (95% CI 1.59-8.06) for the negative CD47 group *vs.* 45.8 (95% CI 0.0-126.4) for the positive CD47 group.

**Figure 3:**
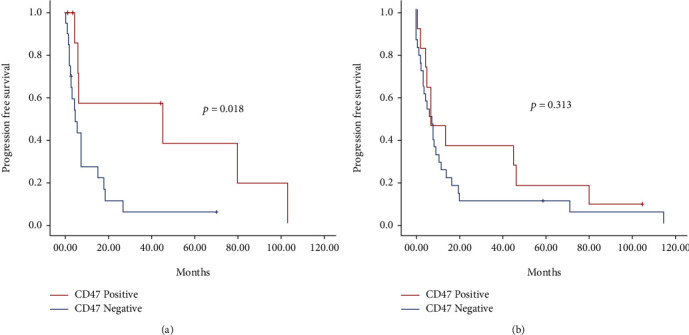
PFS (a) and OS (b) according to CD47 expression in patients with KI‐67 ≥ 40%.

Albeit, these differences did not reach statistical significance when analyzing OS (6.43 (95% CI 2.65-10.22) for the negative CD47 group *vs.* 6.99 (95% CI 0.0-16.64) for the positive CD47 group) (Figures 3(a) and 3(b)).

## 4. Discussion

LNET are a heterogeneous group of tumors associated with poor prognosis and limited treatment options; besides, there are few biomarkers useful in predicting treatment response and prognosis. Some LNET, such as SCLC, display high immunogenicity, a high mutation burden, and other factors that make them favorable for immunotherapy, making immune checkpoint inhibitors (ICIs) a possible breakthrough in the treatment of SCLC and other neuroendocrine tumors [8].

LNET are classified into four histological variants, namely, TC, AC, LCNEC, and SCLC. TC and AC show modifications in the somatic mutation rate and alterations in diverse gene pathway that make them molecularly distinct from SCLC and LCNEC, and different patient subsets exist within each variant of LNET [4]. For example, SCLC can be subcategorized into neuroendocrine-high and neuroendocrine-low subtypes with a different immunological microenvironment. Neuroendocrine-high tumors are characterized by decreased immune cell infiltration; meanwhile, neuroendocrine-low are associated with increased levels of immune cell-related RNA expression. Therefore, neuroendocrine-low patients could more likely respond to immunotherapies [22]. In addition to the presence of heterogeneous population comprised of innate and adaptative immune cells, the expression of specific immune checkpoints is also a crucial immune-suppressing factor in LNET. SCLC tumors present RNA levels for macrophage markers (including SIRP*α*), and their analysis show a positive association between macrophage infiltration and tumor stage [23].

Cancer cells may escape the immune surveillance of macrophages by upregulation of CD47 expression which dampens innate immune response [19, 24]; this assumption makes CD47 inhibition a plausible treatment strategy for patients with LNET [23]. The expression of CD47 mRNA was confirmed in human SCLC cells, SCLC cell lines, and xenografted tumors[23, 25, 26]. To the best of our knowledge, there are no data about its expression on TC, AC, and LCNEC. Furthermore, it is still undetermined the role of CD47 in these tumors. The aim of the present study was to evaluate CD47 as a biomarker of prognosis in 51 patients with LNET.

We confirmed expression of CD47 in 27.5% of our patients with LNET. CD47 expression was apparent in 25% of carcinoid, 25% of atypical, 40% of large cell, and 24% of small-cell tumors. Despite initial expectations, we found that patients with a Ki67 >67% and positive expression of CD47 had a better PFS (45.7 versus 7.8 months; *p* = 0.058). When comparing CD47 expression between NSCLC and LNET, it is evident that CD47 is less frequently expressed in LNET (84% vs. 27.5%) [27]

In our study, we used the Ki-67 labelling index to classify samples in two categories: low grade (TC and AC) and high grade (LCNEC and SCLC) [28]. As expected, low-grade LNET patients had better PFS and OS than high-grade LNET patients. Interestingly, patients with high-grade LNET and CD47 positive expression showed better PFS; to the best of our knowledge, this observation has never been reported in lung cancer. This “paradox” might be explained by pathways regulated by CD47 other than SIRP. However, a reliable definitive explanation of how higher expression of CD47 in LNET might be associated with better PFS and OS remains to be understood.

It has been reported in several malignancies, including NSCLC, that the increased expression of CD47 is associated with a worse prognosis [27]. The physiopathological mechanism of this association appears intuitive, since higher levels of CD47 will more efficiently inhibit SIRP*α* on macrophages and therefore the tumor will evade innate immune response. However, it should be underscored that CD47 also regulates other pathways and has other extracellular, membrane, and intracellular ligands. The effect of inhibiting CD47 in signaling pathways, other than SIRP*α*, is less studied and might be different according to tumor type [26]. For example, CD47 could also interact with thrombospondin-1 (TSP-1), which is an extracellular ligand secreted by vascular and inflammatory cells; TSP-1 regulates cell motility, proliferation, and differentiation of some tumor cell lines *in vitro* [29]. Binding of TSP-1 to CD47 inhibits angiogenesis, induces several death pathways, and modifies the immune response. Ligation of CD47 by TSP-1 inhibits the activation of soluble guanylyl cyclase, prevents VEGFR2 activation, and suppresses NO-dependent proangiogenic pathways [30, 31]. Furthermore, TSP-1 signaling through CD47 improves angiogenic function and upregulates the expression of cell cycle promoters [32]. In prostate cancer cells, the lack of CD47 or blocking TSP-1-CD47 signaling promotes angiogenesis and enhances vascular integrity, leading to accelerated tumor progression [33]. In addition, incubation of breast cancer cells with a CD47 agonist peptide (4N1K) derived from TSP-1 induces apoptosis in a dose-dependent manner [34]. In SCLC cells, TSP-1 participates in *α*3*β*1 integrin adhesion and neurite-like differentiation and prevents their proliferation [35]. Moreover, TSP-1 has a role in immune response; binding of TSP-1 to CD47 promotes macrophage recruitment, induces T cell adhesion, and acts as an autocrine negative regulator for dendritic cell activation [36–38]. Therefore, an increased expression of CD47, as we found in this work, could promote TSP-1-CD47 signaling, inhibit angiogenesis, trigger apoptosis of tumoral cells, and modulate the immune response in order to promote macrophage recruitment.

Results of this study failed to reveal a correlation between the expression of CD47 and poor prognosis of LNET patients. Likewise, the Human Protein Atlas portal (http://www.proteinatlas.org), a Swedish-based program initiated in 2003 with the aim to map all the human protein in cells, tissues, and organs, showed that CD47 RNA levels could be a favorable prognostic marker for the survival of patients with thyroid papillary carcinoma. The portal presents an interactive survival scatter plot and a survival analysis using data from the Cancer Genome Atlas (https://www.proteinatlas.org/ENSG00000196776-CD47/pathology/thyroid+cancer). Patients were classified into two expression groups (“low” and “high”), and the correlation between expression levels and patient survival was examined. Interestingly, patients with a high CD47 expression presented higher survival rates than patients with low CD47 expression. Interestingly, thyroid papillary tumors show focal staining for neuroendocrine markers such as chromogranin and synaptophysin. [39]. These data suggest that hormonal disturbances like those presented in LNET and hormone-producing endocrine cells could have a specific effect in the role of CD47.

Limitations of our study are many and should be considered before making conclusions. First, the retrospective nature of our work is a significant disadvantage. Second, a reliable cut-off point for evaluating the expression of CD47 is still undetermined. Third, we did not evaluate if CD47 was a biomarker of response to anti-CD47 drugs, such as magrolimab (Gilead Sciences); therefore, it remains a possibility that CD47 is a useful biomarker for predicting response to immunotherapy and this should be further evaluated by prospective trials. Fourth, the lack of enough tumor tissue limited us to analyze tumor cell infiltrate. Due to the limitations of our study, we consider that our results should be confirmed by larger, ideally prospective, trials.

## 5. Conclusion

Contrary to what happens in a wide range of hematologic and solid tumors, a higher expression of CD47 in patients with LNET patients is associated with a better progression-free survival, especially in patients with a Ki‐67 ≥ 40%. The real role of CD47 and SIRP*α*-CD47-TSP-1 axis remains to be fully explained in LNET.

## Figures and Tables

**Table 1:** General characteristic of the entire population according to CD47 expression (positive *vs.* negative).

Characteristics	Categories	ALL % (*n*)	CD47- % (*n*)	CD47+ % (*n*)	*p* value
		(*N* = 51)	(*N* = 37)	(*N* = 14)	
Gender	Female	59 (30)	66.7 (20)	33.3 (10)	
	Male	41 (21)	81 (17)	19 (4)	0.346
	Median (±SD)				
Age	<60 years	54.9 (28)	82.1 (23)	17.9 (5)	
	≥60 years	45.1 (23)	60.9 (14)	39.1 (9)	0.120
Smoking history	Nonsmoker	39.2 (20)	27.5 (14)	30 (6)	
	Smoker	60.8 (31)	77.5 (23)	25.8 (8)	0.758
ECOG PS	0-1	78.4 (40)	70 (28)	30 (12)	
	2+	21.6 (11)	81.8 (9)	18.2 (2)	0.705
Disease stage	IIIB	27.5 (14)	71.4 (10)	28.6 (4)	
	IV	72.5 (37)	73 (27)	27 (10)	1.0
Histological grade	Carcinoid	15.7 (8)	75 (6)	25 (2)	
	Atypical	7.8 (4)	75 (3)	25 (1)	
	Large cells	19.6 (10)	60 (6)	40 (4)	
	Small cells	56.9 (29)	75.9 (22)	24.1 (7)	0.804
Lung metastases	Absent	88.2 (45)	73.3 (33)	26.7 (12)	
	Present	11.8 (6)	66.7 (4)	33.3 (2)	0.661
Bone metastases	Absent	84.3 (43)	72.1 (31)	27.9 (12)	
	Present	15.7 (8)	75 (6)	25 (2)	1.0
Pleural effusion	Absent	78.4 (40)	67.5 (27)	32.5 (13)	
	Present	21.6 (11)	90.9 (10)	9.1 (1)	0.251
CNS metastases	Absent	72.5 (37)	73 (27)	27 (10)	
	Present	27.5 (14)	71.4 (10)	28.6 (4)	1.0
Liver metastases	Absent	80.4 (41)	68.3 (28)	31.7 (13)	
	Present	19.6 (10)	90 (9)	10 (1)	0.250
Adrenal metastases	Absent	88.2 (45)	73.3 (33)	26.7 (12)	
	Present	11.8 (6)	66.7 (4)	33.3 (2)	0.661
CD47	Positive	72.5 (37)			0.771
	Negative	27.5 (14)			
Ki-67	5-19%	17.6 (9)	77.8 (7)	22.2 (2)	
	20-39%	5.9 (3)	66.7 (2)	33.3 (1)	
	40-80%	19.6 (10)	60 (6)	40 (4)	
	50-100%	56.9 (29)	75.9 (22)	24.1 (7)	

**Table 2:** Univariate and multivariate analysis for factors associated with PFS.

		Progression-free survival			
		Median, 95% CI	*p*	HR (95% CI)	*p*
Gender	Female	18.2 (0-43.1)			
	Male	7.7 (4.9-10.5)	0.067		
Age	<60 years	10.3 (5.9-14.7)			
	≥60 years	18.8 (0.4-37.2)	0.415		
Tobacco smokers	Nonsmoker	27.3 (3.0-51.7)			
	Smoker	6.2 (3.8-8.6)	0.163		
ECOG PS	0-1	10.3 (0-20.9)			
	2+	4.6 (N/R)	0.393		
Disease stage	IIIB	N/R (N/R)			
	IV	6.5 (4.3-8.6)	0.002	1.6 (0.7-4.0)	0.237
Histological subtype	Carcinoid	NA			
	Atypical				
	Large cell				
	Small cell				
Lung metastases	Absent	10.3 (0.2-20.4)			
	Present	3.4 (1.1-5.7)	0.349		
Bone metastases	Absent	7.8 (1.2-14.4)			
	Present	10.3 (3.6-17.0)	0.579		
Pleural effusion	Absent	16.9 (4.1-29.7)			
	Present	3.4 (0.0-6.8)	<0.001	9.4 (2.3-38.5)	0.02
CNS metastases	Absent	16.9 (6.8-27.1)			
	Present	6.5 (4.3-8.6)	0.033	5.2 (1.4-19.3)	0.013
Liver metastases	Absent	15.7 (0.0-32.3)			
	Present	6.0 (0.0-11.9)	0.018	3.1 (1.0-9.6)	0.047
Adrenal metastases	Absent	10.3 (3.4-17.2)			
	Present	7.8 (7.6-8.1)	0.593		
CD47	Negative	7.8 (4.9-10.7)			
	Positive	45.7 (0.0-115.3)	0.059	0.7 (0.2-2.5)	0.620

**Table 3:** Univariate and multivariate analysis for factors associated with OS.

		Overall survival			
		Median, 95% CI	*p*	HR (95% CI)	*p*
Gender	Female	14.1 (0.0-29.3)			
	Male	5.1 (0.0-11.8)	0.012	0.8 (0.3-1.9)	0.695
Age	<60 years	8.0 (3.8-12.1)			
	≥60 years	14.0 (1.9-26.0)	0.801		
Tobacco smokers	Nonsmoker	19.7 (0.1-39.4)			
	Smoker	7.6 (4.5-10.7)	0.036	1.7 (0.7-4.2)	0.233
ECOG PS	0-1	16.6 (7.6-25.6)			
	2+	3.5 (1.1-5.9)	<0.001	11.1 (3.8-32.1)	<0.001
Disease stage	IIIB	71.5 (N/R			
	IV	6.9 (4.1-9.8)	0.001	1.2 (0.7-2.2)	0.429
Histological subtype	Carcinoid	N/R (N/R)			
	Atypical	30.4 (5.6-55.2)			
	Large cell	6.9 (2.6-11.3)			
	Small cell	6.8 (1.2-12.5)	0.003	1.3 (0.8-2.0)	0.225
Lung metastases	Absent	10.8 (2.9-18.7)			
	Present	6.9 (2.3-11.6)	0.961		
Bone metastases	Absent	8.0 (5.9-10.8)			
	Present	14.0 (2.8-25.2)	0.751		
Pleural effusion	Absent	14.1 (1.6-26.6)			
	Present	6.9 (2.3-11.6)	0.003	3.2 (1.1-9.0)	0.026
CNS metastases	Absent	19.7 (6.8-32.7)			
	Present	5.1 (2.7-7.6)	0.010	2.7 (1.1-6.5)	0.028
Liver metastases	Absent	14.0 (3.1-24.8)			
	Present	6.4 (0.0-14.4)	0.029	2.6 (1.0-6.6)	0.038
Adrenal metastases	Absent	8.0 (4.9-11.0)			
	Present	11.6 (4.7-18.6)	0.798		
CD47	Negative	8.4 (4.6-12.3)			
	Positive	14.0 (0.0-57.0)	0.451	0.835 (0.3-1.9)	0.677

## Data Availability

Generated data is available throughout a reasonable request to the corresponding author.
